# Evaluation of successful treatment in achalasia with timed barium esophagogram: revisiting an old friend

**Published:** 2010-02-25

**Authors:** L Negreanu, BO Popescu

**Affiliations:** Gastroenterology and Neurology Department, University Hospital, BucharestRomania

**Keywords:** achalasia, pneumatic dilation, timed barium, esophagogram, manometry

## Abstract

Achalasia is a rare idiopathic, primary esophageal motility disorder. Pharmacologic, endoscopic and surgical methods are used in its treatment. The efficiency of the treatment is generally based on clinical ‘subjective’ criteria. Manometry, which is the gold standard in diagnostic and in treatment monitoring is not always available, it is costly and it needs expertise. The role of timed barium esophagogram in the evaluation of the patients before and after endoscopic dilation will be discussed in the article. This method is standardized, less costly, and has a good correlation with clinical and manometrical results.

Achalasia is the most commonly seen and the best-known primary esophageal motility disorder. This rare, idiopathic disorder is characterized by a failure of relaxation of the lower esophageal sphincter (LES) and by lack of peristaltic contraction of the esophageal body. In primary or idiopathic achalasia the failure of the deglutitive inhibition is responsible for aperistaltism. This dysfunction is caused by loss of inhibitory nerves and progressive degeneration of ganglion cells containing vasoactive intestinal peptide (VIP) and nitric oxide (NO). Hypertensive LES is thought to result from a combination of the lack of tonic inhibitory nitrergic influence and an unopposed cholinergic activity [1].

Manometry is the gold standard for the diagnosis, but it is available only in expert centers, so typical clinical and radiological manifestations may be sufficient. Barium swallow has long been used to diagnose achalasia. The classic radiological finding associated with achalasia is the ‘bird's beak’ appearance of the distal esophagus. Other radiological findings include an air–fluid level or a tortuous, sigmoid esophagus (especially in long–standing achalasia) [1].

Current treatments include pharmacologic, instrumental and surgical methods but none restores the peristalsis of the esophageal body or the normal pressure of the lower esophageal sphincter. Medical therapy has a limited and transitory effect and it is used only as a bridge to endoscopy or surgery. The European attitude favors endoscopic methods whilst surgery is preferred in the US [2].

Both endoscopic (pneumatic dilation and botulinum toxin injection) and surgical methods have as end points the relief of symptoms and the achievement of esophageal emptying [1, 2].

The success rate of the Rigiflex pneumatic dilatations vary from 50% to 93% [2]. This variability was attributed to differences in technique, although the data in the literature is conflicting. The decision to stop therapy is usually based on symptomatic relief. This ‘clinical approach’ assumes that symptomatic improvement is associated with physiologic improvement, which is not always the case. Eckardt and co–workers compared a detailed score of clinical symptoms before and after treatment and observed that symptomatic improvement may not accurately reflect optimal or complete esophageal emptying [1, 3] (See Table 1).

**Table 1:** Criteria of treatment efficiency in achalasia [1]

Clinical	Eckardt score < 3
Radiology	Esophageal diameter < 3cm
	Barium column height < 1cm, 5 minutes after ingestion
	Diameter of esogastric junction of 8 to 10mm
Manometry	Pressure of LES < 10mmHg

The timed barium esophagogram (TBE) was firstly used by Oliveira and co–workers in 23 achalasia patients studied retrospectively before and after pneumatic dilatation or *Clostridium botulinum* toxin injection [4]. The method is a further development of the barium swallowing, introducing functional and dynamic dimensions to the classic radiological assessment. In their original paper, the authors stated that their purpose was ‘to define a simple, noninvasive, and widely available barium technique that could serve as an objective measure of esophageal baseline and post–therapy emptying in achalasia patients’. 

The original technique described by Oliveira was to obtain upright frontal spot films of the esophagus at 1, 2 and 5 minutes after the ingestion of 100–200 ml of low–density (45% weight in volume) barium sulfate (volume of barium determined by patient tolerance). The authors concluded that timed barium swallowing is a simple and reproducible technique and that both qualitative assessment and estimated change in an area based on height–times–width measurements of the barium column are accurate methods of estimating esophageal emptying [4].

After the initial description, the method (or its variants) was used in treated and non-treated achalasia patients. Some advantages of the TBE are: it is a diagnostic modality that could be used outside the tertiary centers, or when manometry is not available [5]. In our opinion, this is an excellent diagnostic tool, which offers acceptable accuracy and it is largely available. In addition, it can be utilized before the use of more advanced and more costly technology [5].

Two studies by Vaezi and coll. discuss the predictive value of the esophageal emptying on TBE before and after treatment of achalasia patients. 

In the first study [6], there was a 73% concordance between the degree of symptom improvement and degree of esophageal emptying by barium esophagogram (results of 53 pneumatic dilatations in 37 achalasia patients). The patients reporting only a slight symptom improvement (<50%) usually showed (17/19 patients, 89%) a poor esophageal emptying on TBE. The study also showed that up to 30% of dilated patients still had poor esophageal emptying of barium (<50%) although they reported 90–100% symptom improvement [6]. The detractors of the TBE find a weakness of the method, although, in fact, this is not a pitfall but a quality. 

Starting from the hypothesis that the group of patients with poor esophageal emptying would have an earlier relapse of their symptoms when followed on long term, the same team conducted a new study [7]. A comparison between the patients with poor esophageal emptying (discordant group) and the patients whose symptom resolution correlated with marked improvement in esophageal emptying (concordant group) was realized. The primary end–point was to determine if the timed barium esophagogram is a better predictor of long–term treatment success (mean follow–up of 6 years) after therapy, than symptom assessment alone. The results confirmed the predictive value of the TBE emptying: 77% of the patients from the concordant group were still in symptomatic remission while none of the discordant group achieved long–term remission. In fact, (90%) patients with discordant findings failed therapy within one year of pneumatic dilations while the failure rate was constantly and slowly decreasing over time for the concordant group. Furthermore, patients in the concordant group, available for follow–up, continued to have complete esophageal emptying as well as long–term symptomatic remission [7]. 

In the light of the original and follow–up studies, the TBE can be considered a predictor of a poor long–term outcome if patients continue to have post pneumatic dilation barium retention. Based on the TBE results a closer follow–up and a more aggressive further therapy might be offered to these patients in order to ensure long–term remission. A new dilatation during the same hospitalization, a more flexible and aggressive dilatations program or surgery referral based on TBE is a very feasible approach [5].

Is there any real weakness of the technique? In a recent study from Sweden, which shows good results for the static parameters of the TBE (precise and reproducible); the esophageal emptying (dynamic) was considered inaccurate for the evaluation due to poor reproducibility [8]. Possibly these more pessimistic results are related to the great heterogeneity of the group of 21 patients: 16 had a previously diagnosed achalasia and five had a newly established diagnosis; in nine patients one or two balloon dilatations were previously made and seven have suffered a Heller myotomy. The authors propose a further evaluation of the TBE in the pre– and post–operative evaluation of newly diagnosed achalasia patients who will subsequently be randomized to either surgery or endoscopic pneumatic dilatation [8].

On the other hand, a good correlation between post therapy reduction in LOS pressure and barium column height was shown in achalasia patients undergoing pneumatic dilation by the same team of Vaezi and al [9], so this is another argument for the use of TBE in the patient's response to therapy evaluation. 

Esophageal manometry is probably the best technique for the evaluation of the response after pneumatic dilation but due to the cost and lack of availability in many centers, post–procedure manometry is not offered to all patients. The post–dilatation evaluation is not done by manometry not even in rich centers in Europe; we have examples in which manometry is done the next morning after procedure or centers where the result is appreciated a month after the procedure by radiology (especially in the US where the costs are somehow differently judged).

The development of a method that could objectively evaluate the therapy results seemed natural and the results of the TBE are convincing and reliable. Its routine use will simplify the diagnosis, the treatment evaluation and the follow-up of achalasia patients.
